# Tri-Ponderal Mass Index vs. Fat Mass/Height^3^ as a Screening Tool for Metabolic Syndrome Prediction in Colombian Children and Young People

**DOI:** 10.3390/nu10040412

**Published:** 2018-03-27

**Authors:** Robinson Ramírez-Vélez, Jorge Enrique Correa-Bautista, Hugo Alejandro Carrillo, Emilio González-Jiménez, Jacqueline Schmidt-RioValle, María Correa-Rodríguez, Antonio García-Hermoso, Katherine González-Ruíz

**Affiliations:** 1 Centro de Estudios Para la Medición de la Actividad Física CEMA, Escuela de Medicina y Ciencias de la Salud, Universidad del Rosario, Bogotá 111221, Colombia; jorge.correa@urosario.edu.co; 2 Grupo GRINDER, Programa de Educación Física y Deportes, Universidad del Valle, Santiago de Cali 76001, Colombia; hugo.carrillo@correounivalle.edu.co; 3 Grupo Interdisciplinario de Estudios en Salud y Sociedad (GIESS), Institución Universitaria Escuela Nacional del Deporte, Santiago de Cali 76001, Colombia; 4 Departamento de Enfermería, Facultad de Ciencias de la Salud, Avda. De la Ilustración, 60, University of Granada, 18016 Granada, Spain; emigoji@ugr.es (E.G.-J.); jschmidt@ugr.es (J.S.-R.); macoro@ugr.es (M.C.-R.); 5 Grupo CTS-436, Adscrito al Centro de Investigación Mente, Cerebro y Comportamiento (CIMCYC), University of Granada, 18071 Granada, Spain; 6 Laboratorio de Ciencias de la Actividad Física, el Deporte y la Salud, Facultad de Ciencias Médicas, Universidad de Santiago de Chile, USACH, Santiago 7500618, Chile; antonio.garcia.h@usach.cl; 7 Grupo de Ejercicio Físico y Deportes, Vicerrectoría de Investigaciones, Universidad Manuela Beltrán, Bogotá 110231, Colombia; katherine.gonzalez@docentes.umb.edu.co

**Keywords:** adiposity, fat mass, tri-ponderal mass index, fat mass index, metabolic syndrome, children

## Abstract

Tri-ponderal mass index (TMI) and fat mass index (FMI) have been proposed as alternative approaches for assessing body fat since BMI does not ensure an accurate screening for obesity and overweight status in children and adolescents. This study proposes thresholds of the TMI and FMI for the prediction of metabolic syndrome (MetS) in children and young people. For this purpose, a cross-sectional study was conducted on 4673 participants (57.1% females), who were 9–25 years of age. As part of the study, measurements of the subjects’ weight, waist circumference, serum lipid indices, blood pressure and fasting plasma glucose were taken. Body composition was measured by bioelectrical impedance analysis (BIA). The TMI and FMI were calculated as weight (kg)/height (m^3^) and fat mass (kg)/height (m^3^), respectively. Following the International Diabetes Federation (IDF) definition, MetS is defined as including three or more metabolic abnormalities. Cohort-specific thresholds were established to identify Colombian children and young people at high risk of MetS. The thresholds were applied to the following groups: (i) a cohort of children where the girls’ TMI ≥ 12.13 kg/m^3^ and the boys’ TMI ≥ 12.10 kg/m^3^; (ii) a cohort of adolescents where the girls’ TMI ≥ 12.48 kg/m^3^ and the boys’ TMI ≥ 11.19 kg/m^3^; (iii) a cohort of young adults where the women’s TMI ≥ 13.21 kg/m^3^ and the men’s TMI ≥ 12.19 kg/m^3^. The FMI reference cut-off values used for the different groups were as follows: (i) a cohort of children where the girls’ FMI ≥ 2.59 fat mass/m^3^ and the boys’ FMI ≥ 1.98 fat mass/m^3^; (ii) a cohort of adolescents where the girls’ FMI ≥ 3.12 fat mass/m^3^ and the boys’ FMI ≥ 1.46 fat mass/m^3^; (iii) a cohort of adults where the women’s FMI ≥ 3.27 kg/m^3^ and the men’s FMI ≥ 1.65 kg/m^3^. Our results showed that the FMI and TMI had a moderate discriminatory power to detect MetS in Colombian children, adolescents, and young adults.

## 1. Introduction

Metabolic syndrome (MetS) is a set of metabolic abnormalities that are risk factors for cardiovascular disease (CVD), diabetes mellitus type 2 (DM-2), and atherosclerosis [1]. Central obesity and high blood pressure are frequent components of MetS, which are often associated with insulin resistance and metabolic disorders [2]. Thus, MetS is a strong, independent predictor of all-cause and CVD mortality [3]. In the cross-sectional Insulin Resistance Atherosclerosis Study (IRAS Study), Palaniappan et al. showed that every increase of 11 cm in waist circumference (WC) was associated with an adjusted 80% increased risk of developing MetS within five years [4]. Additionally, in a longitudinal adult population-based cohort study (PAMELA Study), the increased risk of cardiovascular and all-cause mortality of subjects was related to high blood pressure and glucose levels, which are components of metabolic disorder [5].

Obesity, especially visceral fat, is an important link between the components of the MetS, since central obesity is highly prevalent [6]. In Colombia, results from the 2016 Report Card on Physical Activity for Children and Youth [7] showed that 13.4% of children (5–11 years of age) were overweight and 4.1% of the adolescents (12–17 years of age) were obese. This obesity epidemic has been associated with obesogenic factors, such as increased intake of energy-dense diets, a sedentary lifestyle, and low levels of physical activity [8]. As obesity plays a central role in MetS and since the probability of childhood obesity persisting into adulthood is estimated to increase from ~20% at the age of four to 80% by adolescence, the epidemic of pediatric obesity can result in an increased prevalence of hypertension, DM-2, and CVD in adulthood [9]. In this context, the over-accumulation of body fat correlates epidemiologically with various pathophysiological sequelae, including a higher incidence of MetS, which is usually associated with CVD mortality [10]. Thus, epidemiological studies have reported an association of fat distribution and metabolic risk factors, including high blood pressure, hyperglycemia, and dyslipidemia, with a risk of MetS in Colombian children and young people [11,12].

Body mass index (BMI) is based on the finding that adult body weight should be proportional to height squared [13]. However, during childhood and adolescent development, weight is not proportional to height squared, thus undercutting the validity of BMI in adolescents [14]. To rectify the problem that these scaling powers (approximately 2.5–3.5) are inconsistent with BMI (approximately 2) prior to the age of 18 years, BMI z-scores are instead used for children and adolescents [15]. However, BMI percentiles for each age (BMI z-scores) do not consider that both body proportions and body fat levels change during growth in a way that is inconsistent with BMI. Therefore, this approach does not ensure an adequate classification of children and adolescents as normal weight vs. overweight or obese. To replace BMI, Peterson et al. [14] recently tested other body fat indices with the form of mass divided by height^n^, including the tri-ponderal mass index TMI (as kilograms divided by meters cubed), which is based on the ponderal index and the Rohrer Index. Another simple and inexpensive approach for assessing body fat is the fat mass index (FMI, fat mass/(height)^2^, which is a surrogate marker of cardiovascular risk in young adults [16]. The FMI was initially conceived as a component of the BMI and is indeed useful in analyzing BMIs in terms of fat and non-fat (especially for men, though the optimal exponent can be close to one for women and three for children) [17]. However, as highlighted by Burton [18], a theoretically sound index, independent of population statistics, uses the formula of (fat mass in kg)/(*D* × height in m^3^), where *D* is the fat density. Burton [18] asserts that, because *D* is virtually constant, the index may be simplified as (fat mass)/height^3^. Unlike the FMI, this nameless index has the same value for individuals of different sizes that are identical in body composition and proportions. VanItallie et al. [19] proposed an FMI (fat mass/(height)^2^) that considers an individual’s height, while Johnson et al. [20] used a regression-based height exponent of 5.8 to adapt the FMI to nine-year-old children. To date, only one previous study has evaluated the applicability of FMI (fat mass/(height)^2^) to predict MetS, with results confirming its close relationship to the components of MetS [21]. However, there is no consensus as to the cut-off value that can be used to define excess adiposity based on TMI (kg)/height^3^) and FMI (fat mass)/height^3^).

As MetS is acknowledged to be a global public health problem and given the increasing prevalence of METs in the Colombian population, there is a clear need to establish gender-specific TMI and FMI cut-off values for estimating the fat mass and body adiposity dysfunction associated with MetS. Accordingly, the objective of this study was to explore thresholds of TMI and FMI (fat mass)/height^3^) for the prediction of MetS in a large population of Colombian children and youth.

## 2. Methods

### 2.1. Study Design and Sample Population

This research is based on a two-part relatively large-scale study (FUPRECOL) [22] of over 1000 children/adolescents and young adults [23]. We used the data from these two projects, which totaled 4673 participants that were 9–25 years old (*n* = 1047 children 9–12 years; *n* = 1830 adolescents 13–17 years; and *n* = 1796 young adults 18–25 years). All participants and their parents/guardians provided their written, informed consent. Each study was approved by the authorized institutional review boards (UMB N° 01-1802-2013 and UR N° CEI-ABN026-000262) and complied with the Declaration of Helsinki (as revised in Hong Kong in 1989 and in Edinburgh, Scotland, in 2000). The studies also complied with Colombian laws regulating clinical research on human subjects (Resolution 008430/1993 of the Ministry of Health). Exclusion factors included a clinical diagnosis of CVD, DM-1 and 2, pregnancy, the use of alcohol or drugs and in general, the presence of any disease not directly associated with nutrition. Volunteers received no compensation for their participation.

### 2.2. Data Collection

Body weight (kg) was measured using an electric scale (Model Tanita^®^ BC-418^®^ or BF-689^®^, Tokyo, Japan) and height (cm) with a portable stadiometer (Seca^®^ 216, Hamburg, Germany). BMI was calculated as weight (kg)/height (m^2^) [24]. WC (cm) was measured as the narrowest point between the lower costal border and the iliac crest using a metal tape measure (Lufkin W606PM^®^, Parsippany, NJ, USA) in accordance with the guidelines of the International Society for the Advancement of Kinanthropometry [25]. BMI and TMI were calculated as weight (kg)/height (m^2^) and weight (kg)/height (m^3^), respectively, while the BMI z-score was estimated using the World Health Organization Growth Reference from 2007 [24]. For all anthropometric variables, a low technical error measurement was reported (less than 2%).

We determined body fat percentage (BF%) and FMI using bioelectrical impedance analyses (BIA) by whole-body impedance (Tanita Model BC-418^®^, Tokyo, Japan). Detailed information about the BIA technique has been provided in previous studies [26,27]. The FMI was then calculated by dividing each subject’s fat mass (kg) by the cubed value of his/her height (m), as described in Burton [18].

### 2.3. Metabolic Syndrome Diagnosis

Between 6:00 and 9:00 a.m. after 10–12 h of an overnight fast, blood samples were extracted from capillary sampling. Participants were asked not to engage in prolonged exercise 24 h prior to testing. Using enzymatic colorimetric methods, we measured (i) high density lipoprotein cholesterol (HDL-c); (ii) triglycerides; (iii) total cholesterol; and (iv) fasting blood glucose. Over a period of 15 days, the inter-assay reproducibility, estimated by the coefficient of variation, was determined from 16 replicate analyses of eight plasma pools. For all blood samples, we obtained errors of less than 4%.

We measured blood pressure levels on the left arm at the heart level using an automatic device Omron M6 Comfort (Omron^®^ Healthcare Europe B.V., Hoofddorp, The Netherlands). Individuals were seated in a semi-reclined position with arms relaxed and supported, while the midpoint of the arm was positioned at the level of the heart.

In children and adolescents, MetS score was defined in accordance with the updated harmonized criteria of de Ferranti et al. [28] and Magge et al. [29] based on age/gender/height criteria. In young adults, MetS was defined in accordance with the updated harmonized criteria of the IDF [30]. According to the criteria of the IDF, participants were considered to have MetS if they showed three or more of the following: (1) abdominal obesity (WC ≥80 cm in women and ≥90 cm in men); (2) hypertriglyceridemia (≥150 g/dL); (3) low HDL-C (<50 mg/dL in women and <40 mg/dL in men); (4) high blood pressure (systolic blood pressure (SBP) ≥130 mmHg or diastolic blood pressure (DBP) ≥85 mmHg); (5) high fasting glucose (≥100 mg/dL).

We calculated a MetS score that reflected a continuous score of the five MetS risk factors. The MetS score was calculated from the data collected for each participant, based on the IDF [30], Ferranti et al. [28], and Magge et al. [29], with standard deviations using data from the total cohort at baseline. The mean of this continuously distributed MetS was thus zero by definition.

### 2.4. Statistical Analysis

The characteristics of the participants were given as mean values and standard deviation (SD). Independent two-tailed *t*-tests for continuous variables and chi-square (χ^2^) tests for categorical variables were used to examine sex differences and age groupings. The relationships between BMI, FMI, TMI, and MetS were tested by means of partial correlation coefficients. This analysis was adjusted by age and sex. Locally weighted scatterplot smoothing curves were used to illustrate the shape of the relationship between TMI, FMI, and MetS. The results are given for the lineal form. A higher fraction of the explained variance (i.e., R^2^ values) in MetS indicates greater accuracy of the anthropometric indexes.

The area under the curve (AUC), ranging from 0 (worthless test) to 1 (perfect test), was used to predict MetS with the TMI and FMI. AUC has been reported to be a global indicator of diagnostic performance since it represents the ability of the test to correctly classify participants with a high risk of MetS (*p*-values < 0.01 and an AUC > 0.80) [31]. In addition, the positive likelihood ratio LR (+) and the negative likelihood ratio LR (−) were also determined. Cutoff points were chosen based on the highest J-Youden index, which uses the point on the receiver operating characteristic (ROC) curve that is farthest from the line of equality [32]. The analysis of the data was performed with the SPSS statistical software package, version 24.0 (IBM, Chicago, IL, USA) for Windows.

## 3. Results

### 3.1. Study Participants

Table 1 shows the results obtained for the anthropometric variables, body composition, blood pressure, and metabolic biomarker variables. The final sample comprised a total of 4673 children and young people. It was composed of 2001 males (42.9%) and 2672 females (57.1%) with a mean age of 17 ± 4.0 (a minimum age of 9 years and a maximum age of 25 years). In the cohort of children, girls were found to have a significantly lower WC, HDL, and glucose level as well as a greater quantity of body fat (%), FMI, and triglycerides compared to boys (*p* < 0.05).

In the cohort of adolescents, the weight, height, WC, SBP, mean arterial pressure, and glucose level of the girls were significantly lower in comparison to the boys (*p* < 0.05). In contrast, their BMI, TMI, body fat, FMI, total cholesterol, triglycerides, LDL, and HDL were significantly higher (*p* < 0.001).

In the cohort of young adults, women were found to have a lower weight, height, WC, SBP, DBP, MAP, and triglycerides than men (*p* < 0.05). However, they had a higher TMI, body fat, FMI, total cholesterol, LDL, HDL, and glucose level in comparison to the men (*p* < 0.001). It is worth highlighting that, among young adults, the prevalence of MetS was greater in women than in men (*p* = 0.001).

### 3.2. Relationship between BMI, FMI, TMI, and MetS Score

Table 2 shows the partial correlation between BMI, FMI, TMI, and MetS score. Overall, we showed an acceptable to moderate positive correlation with MetS score (all *p* < 0.01).

### 3.3. Association between FMI, TMI, and MetS Score

**Figure 1:**
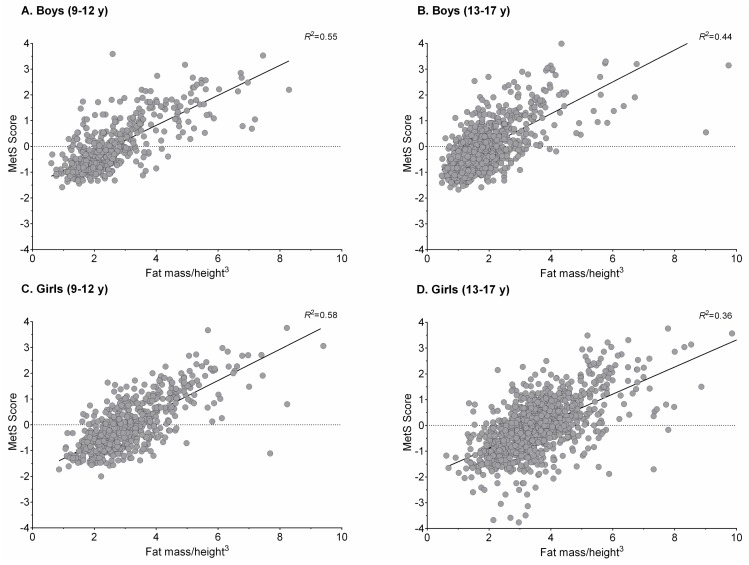

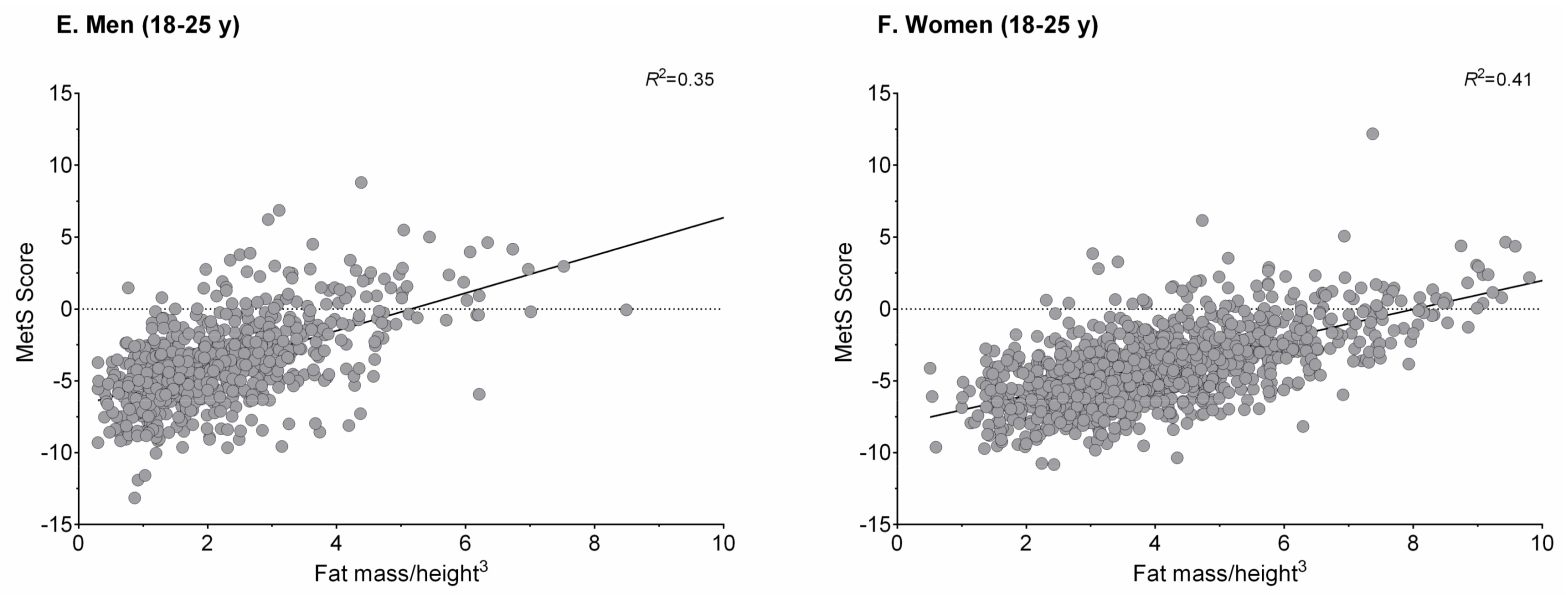
Association between fat mass index and MetS score for sex and age groups. MetS: metabolic syndrome.

**Figure 2:**
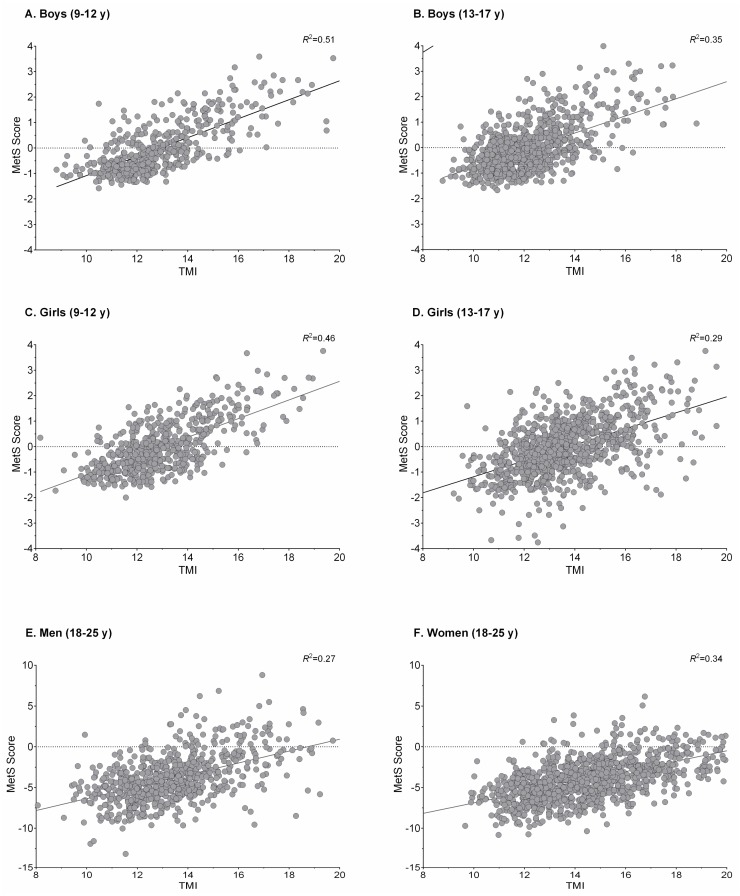
Association between TMI and MetS score for sex and age groups. MetS: metabolic syndrome; TMI: tri-ponderal mass index.

Figure 1 and Figure 2 show plots of the association between FMI, TMI, and MetS score for sex and age groups. As observed, the locally weighted scatterplot smoothing curves provide evidence of a well-defined threshold effect of high/low value indexes on MetS score, which is provided as a continuous outcome.

### 3.4. Optimal Cut-Off Value in the Screening of MetS

The ROC curve analyses for the diagnostic performance of TMI and FMI in identifying a high risk of MetS are shown in Table 3 and Table 4, respectively. The ROC analyses showed that TMI and FMI parameters could be used to detect MetS in Colombian children, adolescents, and young adults.

For the girls in the cohort of children, the TMI cut-off value of 12.13 kg/m^3^ provided a sensitivity of 80%, an LR (+) value of 2.04, a specificity of 61%, and an LR (−) value of 0.33. Regarding the FMI, the cut-off value of 2.59 fat mass/m^3^ provided a sensitivity of 85%, an LR (+) value of 2.05, a specificity of 59%, and an LR (−) value of 0.26. For the boys in the same cohort, the TMI cut-off value of 12.10 kg/m^3^ provided a sensitivity of 85%, an LR (+) value of 2.05, a specificity of 59%, and an LR (−) value of 0.26. The ROC curve for the FMI was also obtained using a cut-off value of 1.98 fat mass/m^3^, which achieved a sensitivity of 82%, an LR (+) of 2.04, a specificity of 60%, and an LR (−) of 0.31.

For the girls in the cohort of adolescents, the TMI cut-off value of 12.48 kg/m^3^ provided a sensitivity of 86%, an LR (+) value of 2.87, a specificity of 70%, and an LR (−) value of 0.20. For the FMI, the cut-off value of 3.12 fat mass/m^3^ provided a sensitivity of 87%, an LR (+) value of 2.55, a specificity of 66%, and an LR (−) value of 0.19. For the adolescent boys in this cohort, the TMI cut-off value of 11.19 kg/m^3^ provided a sensitivity of 93%, an LR (+) value of 3.09, a specificity of 70%, and an LR (−) value of 0.10. The ROC curve for FMI was also obtained using a cut-off value of 1.46 fat mass/m^3^, which achieved a sensitivity of 84%, an LR (+) of 2.10, a specificity of 60%, and an LR (−) of 0.27.

For the women in the young adult cohort, the TMI cut-off value of 13.21 kg/m^3^ provided a sensitivity of 94%, an LR (+) value of 2.81, a specificity of 67%, and an LR (−) value of 0.09. For FMI, the cut-off value of 3.27 fat mass/m^3^ provided a sensitivity of 97%, an LR (+) value of 2.52, a specificity of 62%, and an LR (−) value of 0.08. Regarding the TMI of the men in this cohort, the cut-off value of 12.19 kg/m^3^ provided a sensitivity of 94%, an LR (+) value of 3.11, a specificity of 70%, and an LR (−) value of 0.09. The ROC curve for FMI was also obtained using a cut-off value of 1.65 fat mass/m^3^, which achieved a sensitivity of 93%, an LR (+) of 2.14, a specificity of 57%, and an LR (−) of 0.13.

## 4. Discussion

MetS is considered a worldwide public health problem that increases the risk of cardiovascular morbidity and mortality [10]. In Colombia, the prevalence of MetS was found to be 7.8% and 11.0%, according to the definitions proposed by the Ford et al. and de Ferranti et al., respectively in a population-based sample of schoolchildren [11]. Our study found that the MetS prevalence was higher in girls in the cohorts of children and adolescents (14.6% and 8.1%), while the prevalence was higher in males for young adults. These findings differ from the results of Ruano-Nieto et al. [33], who found that the estimated prevalence of MetS was 8.4% for women and 6.1% for men in a population of Ecuadorian university students. These differences could be explained by the MetS cluster used, the design method, and the target population. Thus, the identification of useful screening tools to detect MetS early in life is of particular interest since this will facilitate more timely and effective interventions in those subjects who are at greater risk.

This study provides gender-specific TMI and FMI reference thresholds as a way of estimating the fat mass and body adiposity dysfunction associated with MetS during childhood and early adulthood. To the best of our knowledge, this is the first evaluation of the predictive capacity of TMI and FMI for MetS. As reflected in the data obtained in our study, both TMI and FMI have a moderate discriminating power for the detection of MetS in Colombian children and youth. This finding supports the diagnostic capabilities of both indices to identify children and young people at a high risk of MetS. This is clinically relevant, since it is known that MetS increases the risk of CVD. 

The FMI (fat mass/m^2^) has been established as a valid predictor of fatness, which is based on fat mass and height in children and adolescents [34,35,36,37]. However, since previous research has indicated that the FMI does not predict adiposity, this research study focused on the prediction capacity of the FMI (fat mass/m^3^) as proposed by Burton [18]. The results of the MetS ROC analysis demonstrated that FMI showed the greatest AUC compared with the TMI for predicting MetS risk in all groups. 

The thresholds established to detect a high risk of MetS in Colombian children and young people were as follows: (i) children cohort: FMI ≥ 2.59 fat mass/m^3^ (girls); FMI ≥ 1.98 fat mass/m^3^ (boys); (ii) adolescent cohort: FMI ≥ 3.12 fat mass/m^3^ (girls); FMI ≥ 1.46 fat mass/m^3^ (boys); (iii) young adults: FMI ≥ 3.27 kg/m^3^ (women); FMI ≥ 1.65 kg/m^3^ (men).

These results are consistent with those of a previous study that compared the BMI and BF% of a population of 1698 Chinese adults and their accuracy in predicting MetS [21]. Their conclusion was that FMI (fat mass/(height)^2^) was the best screening tool for the prediction of MetS [21]. Nevertheless, differences in ethnicity, genetic susceptibility, lifestyle, and age range of the two study populations signify that the data are not directly comparable [38]. Thus, there is a growing interest in proposing cut-off points for body fat levels for the early detection of CVD risk. Future longitudinal studies should be conducted that will lead to a deeper understanding of the role of different levels of adiposity in regard to CVD risk stratification in children, adolescents, and young adults.

Furthermore, it has been found that in adolescents, TMI estimates body fat levels more accurately than BMI [14]. As shown in our study, in addition to being an effective obesity screening tool, TMI can be used to detect MetS among Colombian children and young adults. The thresholds established to detect a high risk of MetS in Colombian children and young people were as follows: (i) children cohort: TMI ≥ 12.13 kg/m^3^ (girls); TMI ≥ 12.10 kg/m^3^ (boys); (ii) adolescent cohort: TMI ≥ 12.48 kg/m^3^ (girls); TMI ≥ 11.19 kg/m^3^ (boys); (iii) young adult cohort: TMI ≥ 13.21 kg/m^3^ (women); TMI ≥ 12.19 kg/m^3^ (men). These results indicate that TMI is a useful indicator for predicting MetS at early ages. However, the differences observed between boys and adolescent boys justify the need to consider sex- and age-specific thresholds as such thresholds are considered in BMI assessments for children and adolescents [39].

Interestingly, among the study cohorts (children, adolescents, and young adults), the greatest AUCs were observed for TMIs of 0.854 for females and 0.814 for males and for FMIs of 0.882 for females and 0.848 for males in the cohort of young adults. This highlights the fact that TMI and FMI are valuable tools that can be used to combat MetS risk in early adulthood. In this same line, previous studies have reported that age- and sex-associated variations of body fat stem from development during growth [40]. According to Rolland-Cachera [41], a developing maturational process and, thus, the absence of a well-defined fat pattern among the children and adolescents in this study could explain the differences in predictive capacity observed between cohorts.

The present study has certain limitations. Firstly, our analysis was of a cross-sectional sample and did not have a longitudinal design. Because the children and adolescents were still growing, the percentiles in height or BF% could vary markedly. Secondly, our study was limited to a well characterized sample of Colombian children and young people. Given that children and adolescents of different races or ethnic groups show important differences in body composition, which mainly occurs in body fat, our reported cut-off values may not be extrapolated to other populations. Nevertheless, this fact does not limit the interesting results obtained. Future population-based studies conducted in other ethnic or racial groups are required to provide TMI and FMI cut-off values for the detection of MetS during childhood and early adulthood. Finally, due to its cross-sectional design, it is not possible to examine whether TMI and FMI are predictors for future MetS. Therefore, further studies investigating their power longitudinally are required.

It should be highlighted that the main strength of our research is that it is the first study to investigate the predictive power of FMI and TMI in relation to MetS in a large population of 4673 subjects and to provide cut-off values for the identification of MetS by FMI and TMI in a population of children and young people. Accordingly, this research has relevant clinical implications in that it permits the early detection of increased cardiometabolic risk at young ages.

## 5. Conclusions

In summary, FMI and TMI were found to have a moderate discriminatory power to detect MetS in a Colombian population of children and young adults. For the first time, reference cut-off values were provided for the detection of MetS by FMI and TMI in children and young people. The reported thresholds could be used in clinical practice to identify Colombian children and youth at high risk of MetS. Furthermore, these results justify the need to incorporate FMI and TMI in daily clinical practice as indicators for predicting MetS and avoiding its associated complications. However, since most clinicians and researchers globally utilize BMI, there would have to be an overall proven increase in usefulness of TMI and FMI in order to convince the healthcare community to adopt these alternate measures. Future studies of different races and ethnic groups are needed to obtain reference values applicable to populations in countries throughout the world.

## Figures and Tables

**Table 1:** Characteristics among a sample from Colombia (mean (standard deviation (SD)) or frequency (%)).

Characteristic	Children 9–12 Years (*n* = 1047)			Adolescents 13–17 Years (*n* = 1830)			Young Adults 18–25 Years (*n* = 1796)		
	Girls (*n* = 582)	Boys (*n* = 465)	*p*-Value	Girls (*n* = 986)	Boys (*n* = 844)	*p*-Value	Women (*n* = 1104)	Men (*n* = 692)	*p*-Value
Anthropometric variable									
Age (years)	10.8 (1.1)	10.7 (1.1)	0.104	14.6 (1.3)	14.7 (1.3)	0.077	21.9 (1.9)	22.6 (1.2)	0.624
Weight (kg)	38.2 (8.8)	37.6 (9.6)	0.353	50.9 (8.6)	53.0 (10.4)	<0.001	58.7 (10.3)	68.9 (12.1)	<0.001
Height (m)	1.43 (0.09)	1.42 (0.10)	0.526	1.55 (0.06)	1.63 (0.10)	<0.001	1.59 (0.05)	1.72 (0.06)	<0.001
WC (cm)	60.1 (7.1)	62.2 (7.7)	<0.001	65.9 (6.8)	67.5 (6.8)	<0.001	71.5 (8.0)	78.2 (8.0)	<0.001
BMI (kg/m^2^)	18.5 (2.8)	18.4 (3.0)	0.312	21.0 (3.0)	19.9 (2.8)	<0.001	23.2 (3.7)	23.1 (3.6)	0.810
BMI z	0.91 (0.4)	1.12 (0.7)	<0.001	0.51 (0.5)	0.39 (0.3)	<0.001	-	-	-
Overweight by BMI/z-BMI *n* (%) *	141 (24.4)	78 (16.9)	0.001	221 (22.5)	82 (9.8)	0.001	236 (21.4)	144 (20.8)	0.724
Obesity by BMI/z-BMI *n* (%) *	51 (8.8)	47 (10.2)	0.001	43 (4.4)	23 (2.7)	0,001	61 (5.5)	33 (4.8)	0.722
TMI (kg/m^3^)	13.0 (1.9)	12.9 (1.9)	0.447	13.6 (1.9)	12.2 (1.7)	<0.001	14.6 (2.4)	13.4 (2.1)	<0.001
Body fat (%)	23.6 (5.8)	19.3 (6.5)	<0.001	25.7 (6.0)	15.1 (5.9)	<0.001	27.0 (7.2)	15.6 (6.5)	<0.001
FMI (fat mass)/height^3^)	3.2 (1.2)	2.6 (1.3)	<0.001	3.6 (1.3)	1.9 (1.1)	<0.001	4.0 (1.7)	2.2 (1.3)	<0.001
Blood pressure									
Systolic blood pressure (mmHg)	109.6 (13.8)	111.0 (13.7)	0.113	110.6 (11.5)	114.4 (14.0)	<0.001	111.2 (11.1)	120.2 (12.9)	<0.001
Diastolic blood pressure (mmHg)	67.1 (8.6)	66.6 (8.9)	0389	69.4 (8.6)	68.9 (9.4)	0.288	71.7 (9.3)	74.1 (11.4)	<0.001
Mean arterial pressure (mmHg)	81.2 (8.7)	81.4 (8.9)	0.797	83.1 (8.2)	84.0 (9.4)	0.020	91.5 (8.9)	97.2 (10.9)	<0.001
Metabolic biomarkers									
Total cholesterol (mg/dL)	151.3 (29.3)	152.1 (30.3)	0.656	148.3 (31.3)	132.9 (30.3)	<0.001	146.3 (33.3)	132.7 (30.2)	<0.001
Triglycerides (mg/dL)	96.0 (60.4)	86.8 (44.7)	0.006	96.7 (50.2)	84.4 (35.8)	<0.001	88.5 (45.3)	93.7 (48.5)	0.020
LDL-C (mg/dL)	86.0 (26.6)	86.6 (30.0)	0.756	84.6 (29.4)	78.6 (35.9)	<0.001	87.9 (26.1)	81.0 (26.0)	<0.001
HDL-C (mg/dL)	48.4 (13.0)	51.5 (13.1)	<0.001	46.9 (11.7)	44.4 (11.2)	<0.001	43.9 (12.8)	39.5 (10.6)	<0.001
Glucose (mg/dL)	83.3 (15.0)	85.3 (16.2)	0.038	80.5 (16.1)	82.3 (15.5)	0.015	86.0 (11.5)	85.5 (11.7)	<0.001
MetS score	-0.12 (0.13)	-0.14 (0.12)	0.008	-0.13 (0.11)	-0.14 (0.09)	0.077	−3.94 (2.66)	−3.90 (2.78)	0.501
Metabolic Syndrome *n* (%) *									
Yes	85 (14.6)	60 (12.9)	0.428	80 (8.1)	56 (6.5)	0.229	82 (7.4)	166 (9.2)	0.001

Continuous variables are reported as mean values (standard deviations (SDs)) and categorical variables are reported as numbers and percentages in brackets. Significant between-sex differences (*t*-tests or * chi-square test χ^2^). WC: waist circumference; BMI: body mass index; TMI: tri-ponderal mass index, FMI: fat mass index; LDL-C: low-density lipoprotein cholesterol; HDL-C: high-density lipoprotein cholesterol.

**Table 2:** Results of the partial correlation analysis between body mass index (BMI), fat mass index (FMI), Tri-ponderal Mass (TMI) and a continuous score of the five MetS.

Group and Variable	MetS Score	TMI (kg/m^3^)	FMI (Fat Mass)/Height^3^)	BMI
**Children 9–12 years (*n* = 1047)**				
BMI	0.534 *	0.938 *	0.942 *	1
FMI (fat mass)/height^3^)	0.522 *	0.911 *	1	
TMI (kg/m^3^)	0.462 *	1		
cMets	1			
**Adolescents 13–17 years (*n* = 1830)**				
BMI	0.455 *	0.942 *	0.882 *	1
FMI (fat mass)/height^3^)	0.427 *	0.846 *	1	
TMI (kg/m^3^)	0.386 *	1		
cMets	1			
**Young adults 18–25 years (*n* = 1796)**				
BMI	0.600 *	0.971 *	0.943 *	1
FMI (fat mass)/height^3^)	0.602 *	0.912 *	1	
TMI (kg/m^3^)	0.554 *	1		
cMets	1			

Analysis adjusted by co-variables: age and sex, * *p* < 0.01. MetS: metabolic syndrome; BMI: body mass index; TMI: tri-ponderal mass index; FMI: fat mass index.

**Table 3:** Parameters of the receiver operating characteristic (ROC) curves analysis for the diagnostic performance of Tri-ponderal Mass (TMI) vs. fat mass index (FMI) in identifying high risk of metabolic syndrome (MetS) according to the Ferranti and Maggie criteria in children and adolescents.

High Risk of MetS			
	Parameter	TMI (kg/m^3^)	FMI (Fat Mass)/Height^3^)
Girls (9–12 years)	AUC	0.674	0.698
	95% CI	0.608–0.740	0.634–0.763
	*p*-value	<0.0001	<0.0001
	J-Youden	0.19	0.18
	Cut-off	12.13	2.59
	Sensitivity (%)	80	85
	Specificity (%)	61	59
	LR (+)	2.04	2.05
	LR (−)	0.33	0.26
Boys (9–12 years)	AUC	0.755	0.752
	95% CI	0.677–0.833	0.676–0.828
	*p* value	<0.0001	<0.0001
	J-Youden	0.17	0.19
	Cut-off	12.10	1.98
	Sensitivity (%)	85	82
	Specificity (%)	59	60
	LR (+)	2.05	2.04
	LR (−)	0.26	0.31
Girls (13–17 years)	AUC	0.684	0.699
	95% CI	0.619–0.748	0.635–0.762
	*p*-value	<0.0001	<0.0001
	J-Youden	0.11	0.13
	Cut-off	12.48	3.12
	Sensitivity (%)	86	87
	Specificity (%)	70	66
	LR (+)	2.87	2.55
	LR (−)	0.20	0.19
Boys (13–17 years)	AUC	0.729	0.745
	95% CI	0.654–0.797	0.675–0.816
	*p*-value	<0.0001	<0.0001
	J-Youden	0.19	0.18
	Cut-off	11.19	1.46
	Sensitivity (%)	93	84
	Specificity (%)	70	60
	LR (+)	3.09	2.10
	LR (−)	0.10	0.27

AUC: area under the curve; CI: confidence interval; LR (+): positive likelihood ratio; LR (−): negative likelihood ratio. MetS: metabolic syndrome; TMI: tri-ponderal mass index; FMI: fat mass index.

**Table 4:** Parameters of the receiver operating characteristic (ROC) curves analysis for the diagnostic performance of Tri-ponderal Mass (TMI) vs. fat mass index (FMI) in identifying high risk of metabolic syndrome (MetS) according to the International Diabetes Federation (IDF) criteria in men and women.

High Risk of MetS			
	Parameter	TMI (kg/m^3^)	FMI (Fat Mass)/Height^3^)
Women (18–25 years)	AUC	0.854	0.882
	95% CI	0.805–0.903	0.840–0.924
	*p*-value	<0.0001	<0.0001
	J-Youden	0.14	0.12
	Cut-off	13.21	3.27
	Sensitivity (%)	94	95
	Specificity (%)	67	62
	LR (+)	2.81	2.52
	LR (−)	0.09	0.08
Men (18–25 years)	AUC	0.814	0.848
	95% CI	0.759–0.869	0.800–0.896
	*p*-value	<0.0001	<0.0001
	J-Youden	0.10	0.15
	Cut-off	12.19	1.65
	Sensitivity (%)	94	93
	Specificity (%)	70	57
	LR (+)	3.11	2.14
	LR (−)	0.09	0.13

AUC: area under the curve; CI: confidence interval; LR (+): positive likelihood ratio; LR (−): negative likelihood ratio. MetS: metabolic syndrome; TMI: tri-ponderal mass index; FMI: fat mass index

## Abbreviations

The following abbreviations are used in this manuscript:

BF	body fat
BIA	bioelectrical impedance analysis
BMI	body mass index
CI	confidence interval
CVD	cardiovascular disease
FUPRECOL	in Spanish: Association between Muscular Strength and Metabolic Risk Factors in Colombia
FMI	fat mass index
HDL-C	high-density lipoprotein cholesterol
IDF	International Diabetes Federation
LDL-C	low-density lipoprotein cholesterol
MetS	metabolic syndrome
SD	standard deviation
TMI	tri-ponderal mass index
WC	waist circumference
